# Supplementary material for: Splice-Junction-Based Mapping of Alternative Isoforms in the Human Proteome
Source: Cell Rep. Author manuscript; Available in PMC 2020 Jan 15. (PMC6961840; doi:10.1016/j.celrep.2019.11.026)
Supplement: 2 [file NIHMS1546469-supplement-2.zip › DF1/PXD006675/CardiacFibroblast/CardiacFibroblast_3_MYCBP2_SLLVPVDNIFNASQGVR.pdf]

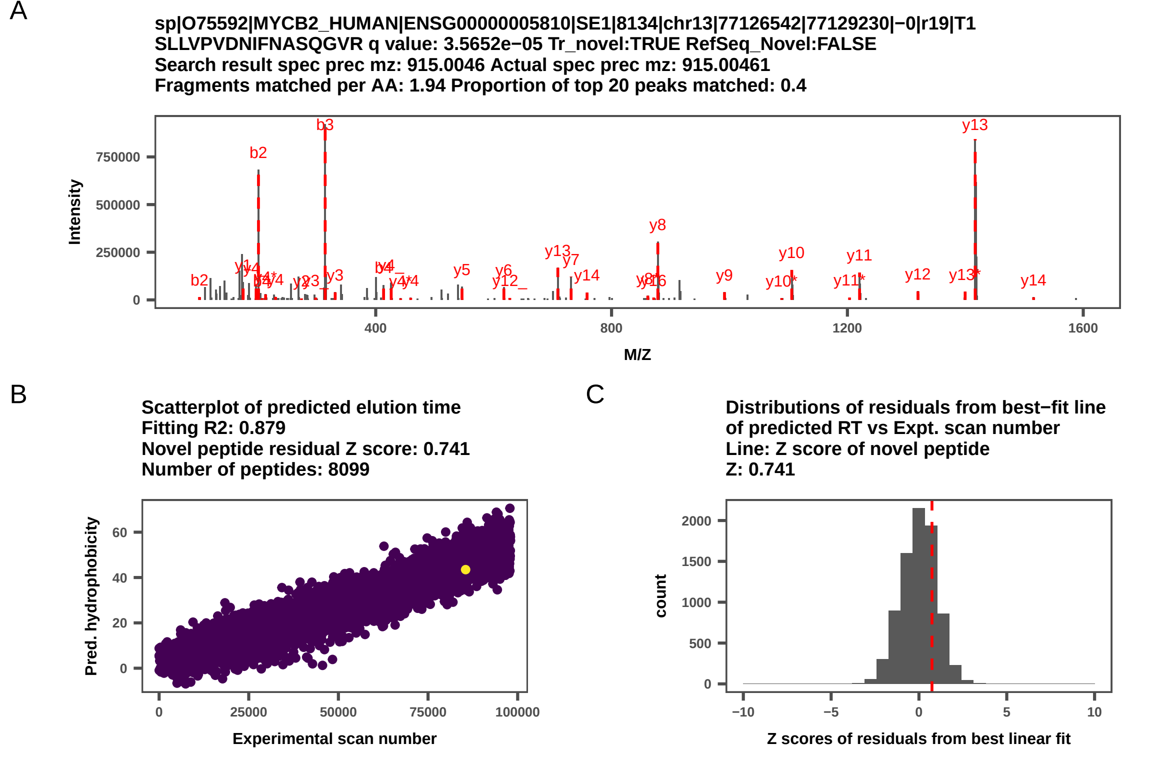
A
sp|O75592|MYCB2_HUMAN|ENSG00000005810|SE1|8134|chr13|77126542|77129230|−0|r19|T1
SLLVPVDNIFNASQGVR q value: 3.5652e−05 Tr_novel:TRUE RefSeq_Novel:FALSE
Search result spec prec mz: 915.0046 Actual spec prec mz: 915.00461
Fragments matched per AA: 1.94 Proportion of top 20 peaks matched: 0.4
Intensity
750000
500000
250000
0
b3
y13
b2
y8
y13
y7
y10
y11
y14
y5
y6
y12_
y9
y10*
y11*
y12
y13*
y14
y3
b2
M/Z
400
800
1200
1600
B
Scatterplot of predicted elution time
Fitting R2: 0.879
Novel peptide residual Z score: 0.741
Number of peptides: 8099
Pred. hydrophobicity
60
40
20
0
0
25000
50000
75000
100000
Experimental scan number
C
Distributions of residuals from best−fit line
of predicted RT vs Expt. scan number
Line: Z score of novel peptide
Z: 0.741
count
2000
1500
1000
500
0
−10
−5
0
5
10
Z scores of residuals from best linear fit
